# Sigmoido-vesical fistula secondary to sigmoid colon cancer presenting as urinary tract infection with *Lactococcus lactis*: A case report

**DOI:** 10.3389/fonc.2023.1054978

**Published:** 2023-03-01

**Authors:** Yanhua An, Qiumei Cao, Yixin Liu, Luping Lei, Dawei Wang, Yanjie Yang, Weijie Kong, Dali An, Dan Liu

**Affiliations:** ^1^ Department of General Practice, Beijing Tongren Hospital, Capital Medical University, Beijing, China; ^2^ Emergency Department, Beijing Tongren Hospital, Capital Medical University, Beijing, China; ^3^ Department of General Surgery, Beijing Tongren Hospital, Capital Medical University, Beijing, China; ^4^ Department of Urologic Surgery, Beijing Tongren Hospital, Capital Medical University, Beijing, China

**Keywords:** sigmoid colon cancer, sigmoido-vesical fistula, urinary tract infection, *Lactococcus lactis*, diabetes mellitus

## Abstract

A colovesical fistula is a pathological communication between the colon and bladder. The symptoms include pneumaturia, fecaluria, and a lower urinary tract infection. The diagnosis is based on clinical symptoms, but the symptoms are not specific. Therefore, confirming the diagnosis is challenging. Urine cultures performed in patients with colovesical fistulas usually show growth of *Escherichia coli* or mixed growth of bowel organisms. Urinary tract infections caused by *Lactococcus lactis* are very rare, as it is rarely considered pathogenic in humans. We report the case of a 70-year-old woman who presented with symptoms of a recurrent urinary tract infection. Urine cultures were positive for *L. lactis*. Abdominopelvic computed tomography (CT) revealed focal thickening of the bladder wall and gas in the bladder. Cystoscopic examination and colonoscopy revealed sigmoid colon cancer and a sigmoido-vesical fistula. Laparoscopic surgical treatment was done. The patient recovered and was discharged 3 weeks later without chemoradiotherapy. On follow-up after 6 months, the patient was asymptomatic and stable. To our knowledge, this is the second reported case of *L. lactis* infection of the urinary tract and the first reported case in adults. *L. lactis* infection usually indicates the presence of serious underlying diseases such as malignancies, uncontrolled diabetes, and organ failure.

## Introduction

Colovesical fistula is a rare complication of various diseases, particularly diverticulitis and neoplasms of the colon and bladder. It is difficult to diagnose because of the atypical symptoms. *Lactococcus lactis* infection is very rare since it is considered nonpathogenic in humans. Here, we present the case of a 70-year-old woman with a recurrent urinary tract infection. Urine cultures revealed *L. lactis*. She was diagnosed with a sigmoido-vesical fistula secondary to sigmoid colon cancer, underwent surgical treatment, and recovered. To our knowledge, this is the first reported case of *L. lactis* infection of the urinary tract in adults.

## Case presentation

A 70-year-old woman was admitted to our hospital due to urinary frequency, urgency, and hematuria of 1 month duration. She also complained of lower abdominal pain and 3 kg weight loss. She presented with a fever for 3 days during this period, with a peak body temperature of 38.3°C. Approximately 10 days prior to admission, she consulted the urology outpatient department and received anti-microbial treatment. Thereafter, only a slight resolution of symptoms was observed.

The patient had a history of type 2 diabetes mellitus for 10 years and was maintained with oral hypoglycemic drugs.

Upon physical evaluation, her vital signs were stable. Enlarged superficial lymph nodes were not palpable. Mild tenderness was observed in the lower abdominal region, without rebound tenderness. No costovertebral angle tenderness was noted.

**Figure 1:**
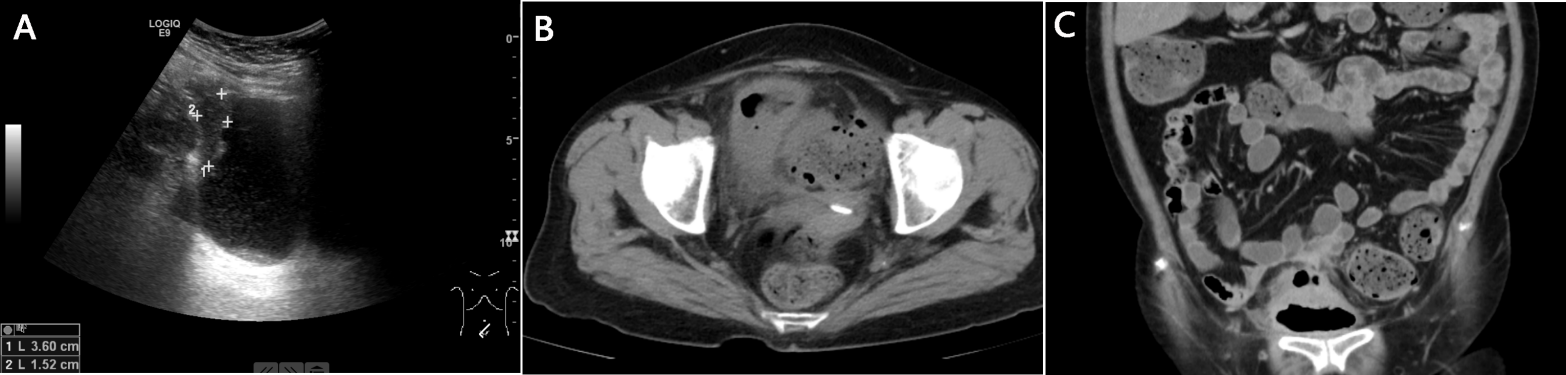
Imaging examinations. **(A)**: Ultrasound examination. The right bladder wall was thickened, and a 3.6 cm × 1.5 cm lesion was revealed; **(B)**: Abdominopelvic computed tomography (CT). Focal thickening of the bladder wall and gas in the bladder were noted; **(C)**: Computed tomography urography (CTU). Gas between the bladder dome and adjacent sigmoid colon, and a thickened bladder and sigmoid colon wall were revealed.

Routine blood tests revealed a normal white blood cell count, hematocrit, and platelet count. Serum C-reactive protein level was increased to 78.38 mg/L (normal range: <10 mg/L). Routine urinalysis showed positive results for red cells (39/high power field), white cells (47/high power field), urine protein, and urine sugar. The fecal occult blood test results were positive. Serum tumor marker analysis revealed that carcinoembryonic antigen (CEA) was mildly elevated to 6.0 ng/ml (normal range: <5 ng/ml). Fasting plasma glucose was 13.6 mmol/L, and glycated hemoglobin A1c (HBA1C) was 8.9%. Urine culture was done thrice, which detected *L. lactis* in two readings. Ultrasound examination showed that the right bladder wall was thickened and revealed a 3.6 cm × 1.5 cm lesion, which was considered likely inflammatory tissue (**Figure 1A**).

**Figure 2:**
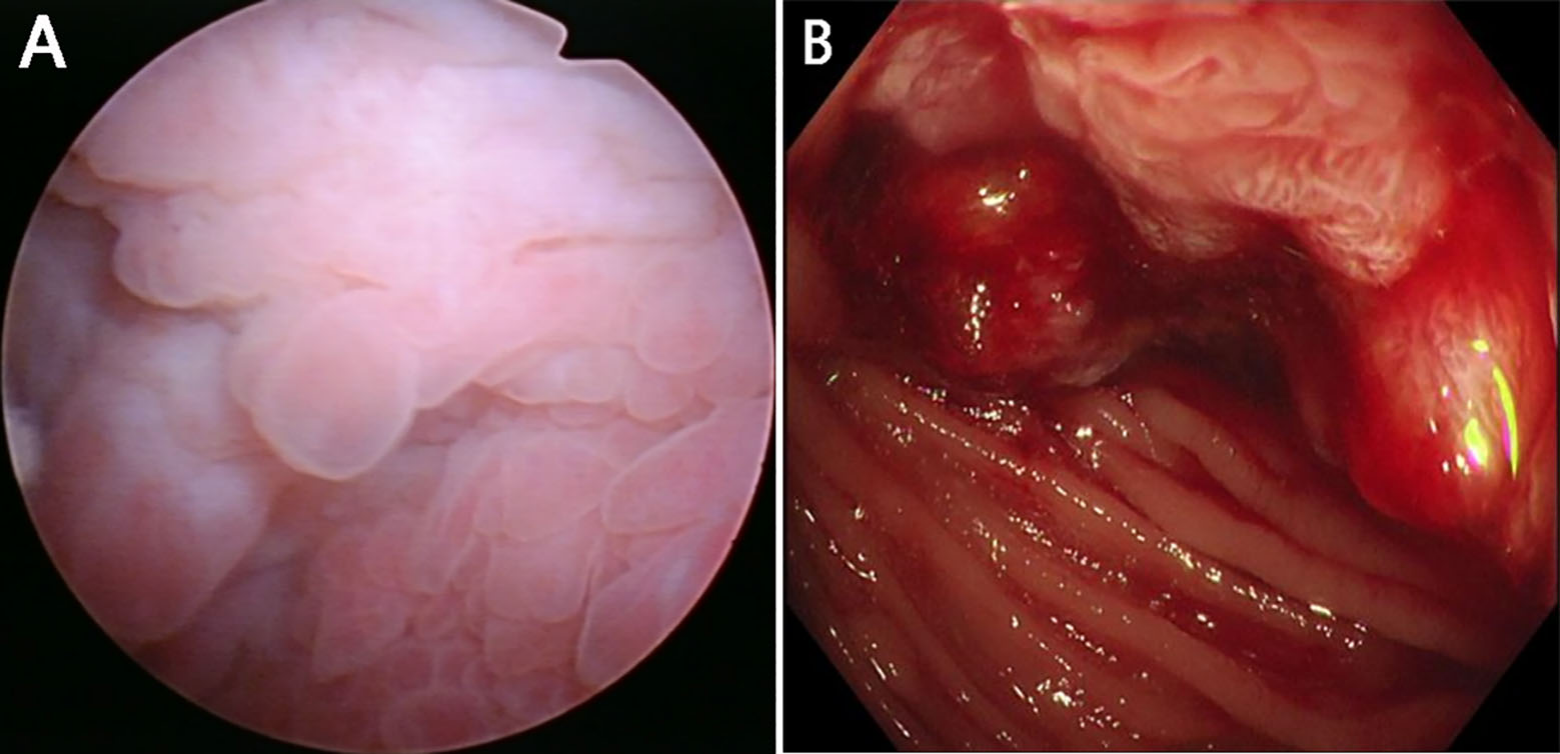
**(A)**: Cystoscopic examination. Changes in the mucosa of the bladder were noted; **(B)**: Colonoscopy. Sigmoid colon cancer was revealed which occupied nearly the entire colon.

On day 10 of admission, the temperature suddenly increased to 39.3°C with worsening lower urinary tract symptoms and lower abdominal pain. Also, foreign bodies were observed in the urine, described as similar to watermelon seeds and tea stems. Abdominopelvic computed tomography (CT) revealed focal thickening of the bladder wall and gas in the bladder (**Figure 1B**). The CT scan also showed thickening of the peritoneum in front of the bladder; however, no significant abnormalities were found in the small intestine or colon. Then computed tomography urography (CTU) was performed, which showed gas between the bladder dome and the adjacent sigmoid colon. These findings were consistent with a sigmoido-vesical fistula (**Figure 1C**). The urologic surgeon performed a cystoscopic examination, which showed turbid urine and a rough bladder wall (**Figure 2A**), but no fistula was found. Colonoscopy was then performed, which revealed sigmoid colon cancer that occupied nearly the entire colon (**Figure 2B**).

The patient was transferred to the general surgery department and underwent laparoscopic surgical treatment. The tumor was visualized to be in the sigmoid colon, where it was observed that the tumor had invaded the entire wall of the colon to the bladder. The colonic wall outside the tumor was adhered to the bladder wall. Enlarged lymph nodes were identified at the root of the mesenterium. No ascites or peritoneal neoplastic dissemination was found during intraoperative exploration.

**Figure 3:**
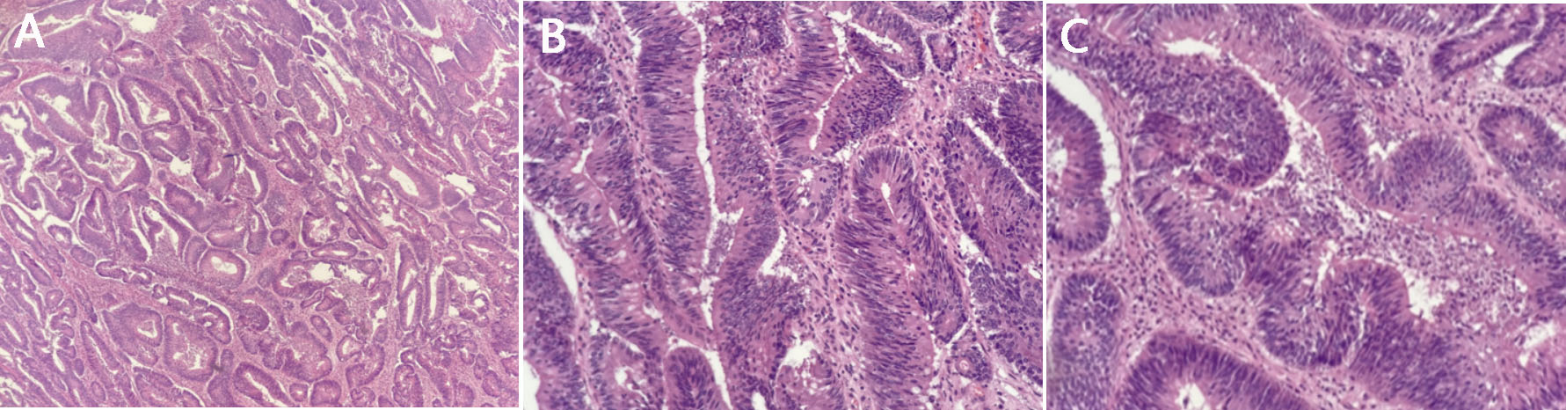
Postoperative pathological outcomes. Highly differentiated tubular adenocarcinoma. **(A)** ×40; **(B)** ×100; **(C)** ×200.

A postoperative pathological examination (**Figure 3**) confirmed persistent, highly differentiated tubular adenocarcinoma (about 7.0 × 4.0 × 4.5 cm in size) accumulating almost the entire colonic wall. The tumor invaded through the muscularis propria into the subserosal adipose tissue. No metastasis was found in 15 peri-colonic lymph nodes. The pathological report also revealed fistulous tract formation between the sigmoid colon and the bladder with severe acute and chronic inflammation in the bladder wall. No tumor invasion was found in the bladder. The tumor was staged as T3N0M0 according to the TNM classification.

The patient recovered and was discharged 3 weeks later without chemo-radiotherapy. Six months after surgery, the patient exhibited normal eating and bowel habits and experienced a weight increase of 1 kg. The blood glucose level was normal and there were no symptoms of a urinary tract infection.

**Figure 4:**
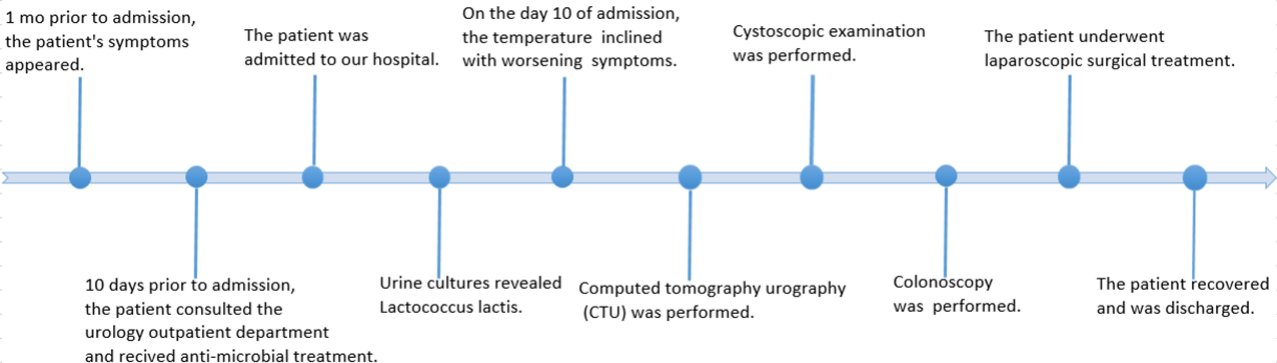
Case timeline.

The case timeline is shown in **Figure 4**.

## Discussion and conclusions

Colovesical fistulas are pathological communications between the colon and bladder (1). Among these, sigmoido-vesical fistulas are the most common (2). Etiological factors include inflammatory diseases, neoplasms of the colon and bladder, pelvic radiation therapy, and traumatic and iatrogenic injuries (3). Diverticulitis is the most common cause, accounting for approximately 65%–79% of cases. The second leading etiology is cancer, contributing 10%–20% of cases, with colonic adenocarcinoma being the most frequent type. Crohn’s disease accounts for 5%–7% of cases (4).

Patients with colovesical fistulas usually present with pneumaturia (50%–85% of cases), fecaluria (51%–68% of cases), and symptoms of lower urinary tract infection (57%–71%), which include frequency, urgency, suprapubic pain, and hematuria (1, 3–5). Our patient presented with urinary frequency, urgency, hematuria, lower abdominal pain, and weight loss.

The diagnosis is based on clinical symptoms; however, the symptoms and signs are not specific. It is challenging to confirm the diagnosis of a colovesical fistula, and it may take months before the condition is recognized. The patient in this case was monitored for almost a month before the diagnosis was confirmed. After admission, poor glycemic control was considered the probable cause of the urinary tract infection; however, the symptoms resolved partly after administration of insulin glargine and intravenous ceftazidime. Further work-up was performed when a sudden worsening of symptoms was observed despite ongoing treatment. A classical presentation of colovesical fistulas is Gouverneur syndrome, characterized by suprapubic pain, frequency, dysuria, and tenesmus (6), which were consistent with the patient. In this case, the fistula was caused by the tumor and repeated inflammatory reactions around it.

Another relevant concern is investigating the specific pathogen. Bacteria that commonly cause urinary tract infections include *Escherichia coli*, Klebsiella, and Enterobacter, which travel from the gastrointestinal tract and perineal area into the urinary tract. Previous studies reported that urine cultures performed in patients with colovesical fistulas showed growth of *E. coli* in approximately 33% of cases and mixed growth of bowel organisms or enterococci in approximately 65% of cases (1, 4). In this case, urine culture was performed three times, which revealed *L. lactis* twice.

Lactococcus is a genus of facultative anaerobic catalase-negative gram-positive intestinal cocci (7). This genus of bacteria is commonly used in manufacturing dairy products and has been investigated for use in the biotechnology industry as a delivery system for vaccines and other therapies (8). Urinary tract infections caused by *L. lactis* are very rare, as it is not considered pathogenic in humans. To our knowledge, this is the second reported case of *L. lactis* infection of the urinary tract and the first reported case in adults (7, 9). A preterm neonate was reported to experience a urinary tract infection caused by *L. lactis* from the gastrointestinal tract after ingestion of the mother’s breast milk (9). *L. lactis* infection occurs more frequently in immunocompromised patients or those with significant underlying conditions such as malignancies, uncontrolled diabetes, and organ failure (10, 11). In our case, the patient had uncontrolled diabetes and sigmoid colon cancer; hence, she was more at risk for opportunistic infections.

CT showed free gas in the bladder, which was initially considered to be produced by bacteria. However, laboratory examinations showed mildly elevated CEA levels and a positive fecal occult blood test, which suggested a possible underlying lesion that should be further investigated. Hence, a clinical history and physical examination, supplemented by appropriate laboratory work-up and imaging, are required to prevent delay in diagnosis.

In conclusion, it is recommended to determine the specific etiology of recurrent urinary tract infections, and colovesical fistulas must be included as a differential diagnosis. If *L. lactis* infection is present, it is essential to identify the underlying diseases, such as malignancies, uncontrolled diabetes, and organ failure.

## Data availability statement

The original contributions presented in the study are included in the article/supplementary material. Further inquiries can be directed to the corresponding author.

## Ethics statement

Written informed consent was obtained from the participant/patient(s) for the publication of this case report.

## Author contributions

YA, QC, and YL were the physicians-in-charge of the patient, reviewed the literature, and contributed to manuscript drafting. LL, DW, and YY reviewed the literature and contributed to manuscript drafting. WK and DA were the patient’s surgeons, analyzed and interpreted the imaging findings. DL performed the cystoscopy and was responsible for the interpretation of the findings. All authors contributed to the article and approved the submitted version.

## References

[1] Pollard SG Macfarlane R Greatorex R Everett WG Hartfall WG. Colovesical fistula. Ann R Coll Surg Engl (1987) 69:163–5. PMC2498444 3631873

[2] Fujii Y Moriguchi Y Taniguchi N. Vesicosigmoidal fistula: Sonographic findings. J Ultrasound Med (2010) 29:993−996. doi: 10.7863/jum.2010.29.6.993 20498474

[3] Granieri S Sessa F Bonomi A Paleino S Bruno F Chierici A. Indications and outcomes of enterovesical and colovesical fistulas: Systematic review of the literature and meta-analysis of prevalence. BMC Surg (2021) 21:265. doi: 10.1186/s12893-021-01272-6 34044862 PMC8157688

[4] Golabek T Szymanska A Szopinski T Bukowczan J Furmanek M Powroznik J. Enterovesical fistulae: Aetiology, imaging, and management. Gastroenterol Res Pract (2013) 2013:617967. doi: 10.1155/2013/617967 24348538 PMC3857900

[5] Daniels IR Bekdash B Scott HJ Marks CG Donaldson DR. Diagnostic lessons learnt from a series of enterovesical fistulae. Colorectal Dis (2002) 4:459–62. doi: 10.1046/j.1463-1318.2002.00370.x 12790920

[6] Vidal Sans J Pradell Teigell J Palou Redorta J Villagrasa Serrano M Banús Gassol JM. Review of 31 vesicointestinal fistulas: Diagnosis and management. Eur Urol (1986) 12:21–7. doi: 10.1159/000472571 3948896

[7] Slaoui A Benmouna I Zeraidi N Lakhdar A Kharbach A Baydada A. Lactococcus lactis cremoris intra-uterine infection: About an uncommon case report. Int J Surg Case Rep (2022) 94:107077. doi: 10.1016/j.ijscr.2022.107077 35461182 PMC9048150

[8] Bahey-El-Din M Gahan CG. Lactococcus lactis: From the dairy industry to antigen and therapeutic protein delivery. Discovery Med (2010) 9:455–61. 20515614

[9] Newby B Ramesh KK. Urinary tract infection in a preterm neonate caused by lactococcus lactis. Can J Hosp Pharm (2014) 67:453–4. doi: 10.4212/cjhp.v67i6.1409 PMC4275143 25548404

[10] Lee MR Huang YT Lee PI Liao CH Lai CC Lee LN. Healthcare-associated bacteraemia caused by leuconostoc species at a university hospital in Taiwan between 1995 and 2008. J Hosp Infect (2011) 78:45–9. doi: 10.1016/j.jhin.2010.11.014 21269734

[11] Shimizu A Hase R Suzuki D Toguchi A Otsuka Y Hirata N. Lactococcus lactis cholangitis and bacteremia identified by MALDI-TOF mass spectrometry: A case report and review of the literature on lactococcus lactis infection. J Infect Chemother (2019) 25:141–6. doi: 10.1016/j.jiac.2018.07.010 30100399

